# Treatment of extramammary Paget’s disease with sirolimus: A case series

**DOI:** 10.1016/j.jdcr.2025.10.021

**Published:** 2025-10-27

**Authors:** Mariah C. Estill, Katherine G. Thompson, Julia M. Kasprzak, Olushola L. Akinshemoyin Vaughn

**Affiliations:** Department of Dermatology, Medical College of Wisconsin, Milwaukee, Wisconsin

**Keywords:** extramammary Paget’s disease, sirolimus, vulvar

## Introduction

Extramammary Paget's disease (EMPD) is a rare, slow-growing intraepithelial adenocarcinoma that can pose a therapeutic challenge to clinicians. EMPD typically presents as pruritic, pink-red plaques distributed at the vulva, penis, scrotum, perineum, perianal skin, or axilla. While most cases of EMPD are noninvasive, long-term follow-up is indicated because EMPD may become invasive, is associated with underlying malignancies, and has a high rate of recurrence.

The standard treatment of EMPD is surgical excision, although extensive surgery can cause permanent anatomical changes and functional impairment, with high recurrence rates.^1^ When surgical therapy is not appropriate or is declined by the patient, radiation therapy and topical imiquimod have been used.^1^^,^^2^ There is limited published research on alternative therapies for EMPD.^3^

While the pathogenesis of EMPD is not completely understood, the Musashi-1- mammalian target of rapamycin signaling pathway is thought to play a role in disease development.^4^ Sirolimus has been shown to inhibit Musashi-1-mammalian target of rapamycin signaling and may be a promising novel therapeutic option for EMPD. A previous study demonstrated efficacy of topical sirolimus in a small cohort of patients.^5^ Here, we report a case series of 4 patients with EMPD who were treated with topical sirolimus.

## Case series

From 2021 to 2023, 4 patients with EMPD (Table I) were treated with topical sirolimus at Froedtert Hospital-Medical College of Wisconsin in Milwaukee, Wisconsin. All patients underwent other standard treatments without success prior to treatment with sirolimus and other alternative therapies. All patients provided verbal informed consent prior to starting sirolimus as an “off-label” treatment.

**Table I:** Clinical characteristics

Case	Age/Sex	Duration of disease prior to sirolimus	Therapies tried prior to sirolimus	Areas of involvement	Subjective improvement on sirolimus	Total duration of sirolimus treatment	Adverse events on sirolimus
1	68/M	2 y	triamcinolone 0.1% ointment, imiquimod for 12 wk ×3 trials	Mons pubis, penile shaft, corona of penis, scrotum	Yes	22 mo	Intermittently irritating to the skin
2	57/F	1 y	right partial vulvectomy, imiquimod 5% cream, betamethasone ointment	labia majora. labia minora and introitus	No	20 wk	Some burning with application of sirolimus
3	68/F	Several year history	clobetasol propionate 0.05% ointment, gentamicin 0.1% ointment, lidocaine 5% ointment	Labia majora, labia minora, perianal areas	Yes	12 wk	Overlying yeast infection
4	69/M	12 y	Mohs surgery	Mons pubis, base of penis, scrotum, perineum	No	8 wk	None

### Case 1

A 68-year-old man presented to clinic with a 2-y history of an erythematous plaque located at the mons pubis with extension onto the penis. Multiple previous biopsies had shown findings consistent with EMPD. He initially received treatment with triamcinolone 0.1% ointment twice daily for 2 weeks and had several 12-week trials of imiquimod 5% cream daily, but minimal improvement on these therapies. The patient had a history of basal cell carcinoma on the left forehead treated with Mohs surgery, but no other personal or family history of malignancy. The patient was up-to-date with age-appropriate cancer screenings including recent colonoscopy without findings concerning for malignancy.

**Fig 1:**
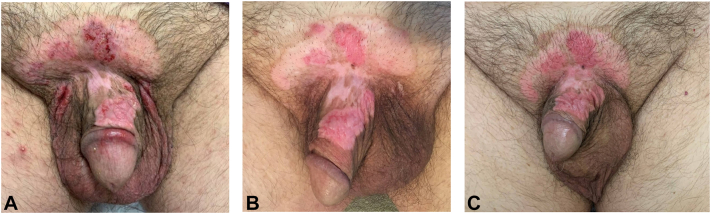
Clinical images of patient 1 at baseline **(A)**, 8 wks on sirolimus twice daily **(B)**, and 16 wks on sirolimus twice daily **(C)**.

On examination, there was a large, well-defined, pink scaly plaque extending across the mons pubis and inferiorly to the dorsal penile shaft, corona, and scrotum, approximately 13 × 9 cm in size. Within this plaque there were foci of dark red macules and erosions (Fig 1, *A*).

Mohs surgery was declined by the patient as he preferred to continue conservatively treating EMPD topically with a goal of maintenance rather than resolution. Imiquimod was discontinued and the patient was started on compounded sirolimus 0.4% ointment twice daily. After 8 weeks on sirolimus, there was significant clinical improvement. The surface area decreased and there was less erythema and cobbled texture of the mons pubis and penile shaft (Fig 1, *B*). After 16 weeks on sirolimus, the patient showed significant but plateaued improvement (Fig 1, *C*). There was a new brown macule located on the mons pubis just superior to the base of the penis, which was biopsied and found to be EMPD with associated pigmentation.

The patient elected to repeat imiquimod in hopes that alternating imiquimod and sirolimus could continue to shrink the area of involvement, but after 12 weeks on imiquimod, he reported interval worsening and irritation with application. He restarted sirolimus for 9 months which resulted in decreased areas of involvement of the scrotum and glans penis and overall stabilization of disease.

He continued compounded sirolimus daily and maintained clinical improvement at follow up 6 months later. The patient was subsequently diagnosed with myelodysplastic syndrome. While on decitabine chemotherapy, he developed neutropenia and used sirolimus sparingly in order to avoid open wounds that might increase his risk of infection. There was no reported skin improvement on decitabine chemotherapy. The patient passed away from complications secondary to myelodysplastic syndrome 6 months later.

### Case 2

A 57-year-old female was seen for evaluation of vulvar tenderness and bleeding. The patient had a history of stage IB grade 1 endometrial cancer and adjuvant vaginal brachytherapy, in remission. She had been diagnosed with EMPD 1 year prior after biopsies found noninvasive Paget’s disease and subsequently underwent a right partial vulvectomy.

Physical exam revealed mottled gray, pink, and white patches on buttocks, posterior and medial labia majora. There was a tender, friable red plaque, approximately 10 cm, on the right medial labium majus. There was also a red erosive-appearing plaque on the right and left medial labia minora and introitus.

Malignancy screening with mammogram and colonoscopy obtained 5 and 3 y previously did not show evidence of underlying malignancy or metastatic disease. Computed tomography (CT) scan of the pelvis showed no evidence of primary or metastatic malignancy. The patient was diagnosed with primary EMPD unrelated to patient’s endometrial adenocarcinoma.

The patient was started on imiquimod 5% cream 3 times weekly and betamethasone ointment twice daily to the vulva, perineum, and vaginal introitus but did not show improvement on these therapies. The patient then started sirolimus 0.3% ointment twice daily, with concentration limited by the compounding pharmacy. She was unable to consistently apply the medication properly due to body habitus with body mass index of 46. She briefly restarted imiquimod but discontinued due to emesis and burning sensation with application. There was some reported burning with application of sirolimus, but it was considered tolerable to the patient. She restarted sirolimus 0.4% ointment twice daily, compounded at a different pharmacy, and augmented betamethasone dipropionate 0.05% ointment twice daily to the vulva, applied at different times. The patient utilized vaginal applicators to assist with application of the medications, but there was still no significant improvement after 5 months, so sirolimus was discontinued.

The patient elected to start a trial of intralesional 5-fluorouracil (0.5 mL at 50 mg/mL). Over a 1-month period, the patient received 5 injections of 5-fluorouracil. Although the injections allowed for some re-epithelization, there was not clinically meaningful improvement. Despite subsequent radiation therapy (6000 cGy in 30 fractions to the vulva), the disease continued to progress. She is scheduled for bilateral vulvectomy with posterior thigh flap.

### Case 3

A 68-year-old female patient presented to clinic with a several year history of a pruritic and painful vulvar eruption. Treatment with clobetasol propionate 0.05% ointment twice daily, gentamicin 0.1% ointment twice daily, and lidocaine 5% ointment every 2 hours as needed had failed to improve symptoms. Findings from a punch biopsy of the “left labia” obtained a few months prior established the diagnosis of EMPD in situ, although invasion could not be ruled out due to the extent of involvement. The patient had no personal history of malignancy. Recent CT of the chest, abdomen, and pelvis was negative for metastatic disease. Mammogram was unremarkable.

**Fig 2:**
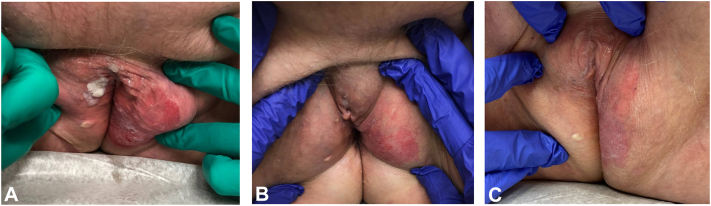
Clinical images of patient 3 at baseline **(A)**, 4 wks on sirolimus twice daily **(B)**, and 8 wks on sirolimus twice daily **(C)**.

Physical exam revealed a dull red patch, approximately 10 × 12 cm extending from the clitoral hood to perineum, with central grayish foci involving the left labium majus, clitoral hood, and right labium majus. There was a white verrucous plaque localized to the right labium majus and eroded plaques on the left labium majus and posterior aspect of the vaginal introitus (Fig 2, *A*).

Management options for EMPD were discussed with the patient. She declined surgical intervention and imiquimod due to concerns for pain with these treatments. She was started on compounded sirolimus 0.4% ointment twice daily, over the counter emollient as needed, and topical lidocaine as a spot treatment for painful areas up to twice daily.

After 4 weeks of treatment with sirolimus, there was significant improvement in erythema and erosions. The plaque previously present on the right labium majus had resolved (Fig 2, *B*). The patient reported intense pruritus over the involved area, which was affecting her mental health. The patient was referred to a quality of life clinic for evaluation of depressed mood and continued sirolimus therapy.

After 8 weeks, there was additional clinical improvement. On exam, there was notable improvement of the eroded thin plaque on the left labium majus (Fig 2, *C*). The patient reported continued itching, most notably at bedtime. She had established care with a behavioral health clinic and planned for psychotherapy for adjustment disorder with anxiety and depressed mood. She continued sirolimus for an 8-week repeat cycle, with pimecrolimus 1% cream twice daily added for management of persistent pruritus. The patient was encouraged to continue care with behavioral health.

After a total of 12 weeks of treatment, the patient reported interval worsening of inflammation, pruritus, and maceration. An overlying yeast infection was suspected. Treatment with sirolimus and pimecrolimus was paused to avoid further potential irritation. She was lost to follow up and died 4 months later by suicide.

### Case 4

A 69-year-old male presented to clinic with a red patch on the left pubic area. He had been diagnosed with EMPD of the scrotum with dermal invasion 10 years prior. At that time, he was treated with Mohs surgery and immediate reconstruction.

Upon reevaluation, physical exam revealed a 3 × 2 cm ill-defined pink plaque located at the base of the penis and 3 other ill-defined pink papules on the mons pubis. A shave biopsy of the left mons pubis demonstrated findings consistent with EMPD in situ. Malignancy screening with CT (chest/abdomen/pelvis), urinalysis, cystoscopy with cytology, and colonoscopy were negative for underlying malignancy. The patient was diagnosed with recurrence of EMPD on the mons pubis, scrotum, and base of the penis and underwent Mohs micrographic surgery using CK7 immunohistochemical staining with 4 stages required for clearance.

Two years later, the patient returned to clinic after developing a persistent pink plaque on the perineum. On physical exam, there was a pink well-demarcated patch on the perineum extending to the perianal area. Based on shave biopsy of the patch, the patient was diagnosed with a second recurrence of EMPD. CT (abdomen/pelvis) showed enhancing soft tissue along the anterior-inferior scrotum without lymphadenopathy in the abdomen or pelvis. After discussion of management options, the patient was started on compounded sirolimus 0.4% ointment twice daily for 8 weeks, but did not report improvement.

Given the involvement of the anal verge, excision with peripheral and deep margin assessment was performed with colorectal surgery in the operating room. Intraoperative frozen sections were performed, and histologic clearance was confirmed after 2 stages. Three years later, the patient remains in observation status without recurrence and has regular follow-up examinations with both dermatology and colorectal surgery for monitoring.

## Discussion

The refractory course and limited effective treatment options for EMPD necessitate reporting of alternative therapies. Although topical sirolimus is only FDA approved for facial angiofibromas, its potential efficacy, suggested by the involvement of the mTOR pathway, has led to its use as a potential “off-label” treatment for EMPD.

The only previously published study on the efficacy of sirolimus for EMPD described a cohort of 4 men with scrotal and penile EMPD. In this study, application of sirolimus 0.4% ointment twice daily for 2 weeks led to a clinically positive response.^5^ All 4 patients in that cohort demonstrated improvement in erythema in clinical photos, as well as in histologic signs of EMPD such as reduced skin thickness and percentage of Paget cells with positive periodic acid-schiff, carcinoembryonic antigen, cell adhesion molecule 5.2 and phosphorylated-S6 ribosomal protein biomarkers. Our case series adds 2 cases of scrotal and penile EMPD treated with sirolimus to the literature, while also contributing 2 cases of vulvar EMPD treated with topical sirolimus. Additionally, our cases demonstrate the clinical response over a longer duration.

In conclusion, 2 patients demonstrated subjective clinical improvement on sirolimus 0.4% ointment twice daily in terms of decreased erythema and size of lesions, although neither patient had complete resolution. Given the paucity of clear effective treatment options, a response rate of 2 out of 4 patients justifies consideration of sirolimus as a palliative treatment option. The limitations of our case series include its small number of patients and lack of standardized outcome measures to assess disease severity. While our limited experience has been promising, larger studies with extended follow-up are necessary to better understand the clinical and histological advantages of sirolimus for EMPD and to identify optimal dosing and frequency.

## Conflicts of interest

None disclosed.
